# Bridge helix and trigger loop perturbations generate superactive RNA polymerases

**DOI:** 10.1186/jbiol98

**Published:** 2008-12-02

**Authors:** Lin Tan, Simone Wiesler, Dominika Trzaska, Hannah C Carney, Robert OJ Weinzierl

**Affiliations:** 1 Department of Life Sciences, Imperial College London, Sir Alexander Fleming Building, Exhibition Road, London SW7 2AZ, UK

## Abstract

**Background:**

Cellular RNA polymerases are highly conserved enzymes that undergo complex conformational changes to coordinate the processing of nucleic acid substrates through the active site. Two domains in particular, the bridge helix and the trigger loop, play a key role in this mechanism by adopting different conformations at various stages of the nucleotide addition cycle. The functional relevance of these structural changes has been difficult to assess from the relatively small number of static crystal structures currently available.

**Results:**

Using a novel robotic approach we characterized the functional properties of 367 site-directed mutants of the *Methanocaldococcus jannaschii *RNA polymerase A' subunit, revealing a wide spectrum of *in vitro *phenotypes. We show that a surprisingly large number of single amino acid substitutions in the bridge helix, including a kink-inducing proline substitution, increase the specific activity of RNA polymerase. Other 'superactivating' substitutions are located in the adjacent base helices of the trigger loop.

**Conclusion:**

The results support the hypothesis that the nucleotide addition cycle involves a kinked bridge helix conformation. The active center of RNA polymerase seems to be constrained by a network of functional interactions between the bridge helix and trigger loop that controls fundamental parameters of RNA synthesis.

## Background

RNA polymerases (RNAPs) are central components of the cellular transcriptional machineries that are targeted by numerous regulatory proteins to fine-tune the expression of genomes in a highly controlled manner. It is therefore important to study the functional properties of RNAPs in order to understand how these are modulated during the various stages of the transcription cycle.

**Figure 1:**
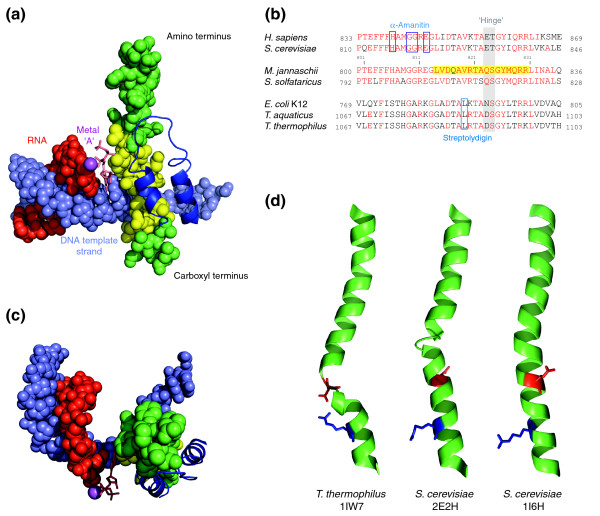
Structure, evolutionary conservation and conformational isomers of the bridge helix. **(a) **Structure of the active site of *Saccharomyces cerevisiae *RNAPII [7] (based on PDB code 2E2H). All structures, except the trigger loop (dark blue) and the rNTP in the insertion site (salmon pink) are shown in the space-filling representation. The bridge helix is green and the region that has been mutagenized for this study is highlighted in yellow. The DNA template strand is in light blue and the nascent transcript red. The Mg^2+ ^ion (metal 'A', magenta) is part of the catalytic site. **(b) **Sequence alignment of representative bacterial [*Escherichia coli *K12 (UniProt/Swiss-Prot accession number P0A8T7), *Thermus aquaticus *(Q9KWU6), *Thermus thermophilus *HB27 (Q72HM6)], archaeal [*Methanocaldococcus jannaschii *(A64430) and *Sulfolobus solfataricus *(NP_341776)] and eukaryotic [*S. cerevisiae *(CAA65619), *Homo sapiens *(NP_000928); RNAPII only] bridge helix sequences. Except for *H. sapiens *and *M. jannaschii*, all other sequences have been selected solely on the basis of the availability of X-ray structures. The numbers on the left and right side of the sequences refer to the amino- and carboxy-terminal amino acid positions of the sequence shown relative to the full-length open reading frame. The numbering of the residues in the *M. jannaschii *sequence is based on the intein-free final product. Amino acid residues identical to the corresponding *M. jannaschii *position are shown in red, the 'hinge' region [11,12] in gray and binding sites of α-amanitin [13,19] and streptolydigin [12,20] are boxed in blue. Note that the location of the hinge region in archaeal and eukaryotic RNAPs is inferred from the position of the kink in bacterial bridge helices. The residues in the *M. jannaschii *sequence that were mutagenized in this study are highlighted with a yellow box. **(c) **Top view (from the amino terminus of the bridge helix) of structure shown in (a). Note the position of the bridge helix relative to the DNA-RNA hybrid. **(d) **Bridge helix conformations as seen in three different X-ray structures. The bridge helix is shown in green in ribbon representation. The species and PDB codes are shown below. Two orthologous residues in each structure, corresponding to D1090 and R1096 in the *T. thermophilus *β' subunit, are shown in stick representation in red and blue, respectively (see text for further discussion of the possible significance of these residues in stabilizing the kinked conformation).

Combined insights from biochemical, genetic and structural studies have led to the unambiguous identification of several structural motifs that participate in the key enzymatic processes of RNAPs (reviewed in [1-4]). Among these, the bridge helix, which is approximately 35 amino acids long, is one of the most prominent features of the active site of all cellular RNAPs (Figure 1a,c). Its primary sequence is highly conserved across the entire evolutionary range, including bacteria, archaea and eukaryotes (Figure 1b and Additional data files 1-17). Structural studies suggest that the bridge helix guides the template DNA strand into the active center and positions the DNA-RNA hybrid relative to the catalytic site. In many RNAP structures the bridge helix is a continuous and gently curved α helix (see, for example, [5-9]). In contrast, in some bacterial RNAP structures the bridge helix is distinctly kinked in the vicinity of the catalytic site [10-12], and recent yeast RNAPII structures have also revealed helical irregularities in more amino-terminal locations [7,13] (Figure 1d). Periodic conversions from the straight to the various kinked bridge helix conformations during each ribonucleotide addition step could, in principle, provide a mechanical basis for translocating the nucleic acid substrates through the active site in single nucleotide steps [5,6,14,15] (Figure 1a,c). Structural changes in an adjacent domain, the trigger loop, are thought to be responsible for influencing the bridge helix conformations [16,17]. Recent models thus emphasize a direct role for the trigger loop in controlling the catalytic functions of RNAPs through conformation-specific contacts with the NTP in the nucleotide insertion site [7,8,18]. The crucial role of the combined bridge helix/trigger loop mechanism in RNAP function is most clearly demonstrated by the inhibitory action of bacterial antibiotics and eukaryotic toxins that block bridge helix and trigger loop movements [12,13,19-21] (Figure 1b).

Although the key domains involved in the translocation of the substrates through the RNAP catalytic site are thus unambiguously identified, there is still a lot of uncertainty concerning the detailed molecular events occurring in the active site during RNA synthesis. This lack of understanding is mostly due to the fact that the current models are based on a small number of 'snapshots' of relatively stable structures that can be studied after crystallization. RNAPs are, however, complex macromolecular machines that undergo multiple conformational changes during the nucleotide addition cycle, which may be too unstable and short-lived to be captured in a rigid crystal structure. We therefore decided to learn more about the functional aspects of the bridge helix/trigger loop mechanism by systematically mutating residues located in critical positions relative to the active site. We employed an archaeal RNAP model system, derived from the hyperthermophilic euryarchaeon *Methanocaldococcus jannaschii*, to reconstitute an active enzyme from separate recombinant subunits *in vitro *[22,23]. Archaeal RNAPs are structurally and functionally very closely related to bacterial and eukaryotic RNAPIIs and thus provide an ideal experimental platform for a structure-function approach that can exploit the large body of data obtained in these mainstream experimental systems [9,24]. The ability to reconstitute recombinant RNAPs *in vitro *allows targeted mutation(s) to be introduced at predetermined locations using efficient DNA cloning and protein expression technologies. This approach, in combination with recently developed robotic methods for assembling recombinant RNAPs in high-throughput format [25], provides the necessary tools for dissecting the functional properties of key RNAP domains at unprecedented resolution. The results obtained shed new light on the role of individual residues and provide evidence for the functional relevance of conformational changes in the active site of RNAPs that are not evident from the previously available structural and genetic data.

## Results

### Bridge helix mutants display a broad spectrum of catalytic activity phenotypes

**Figure 2:**
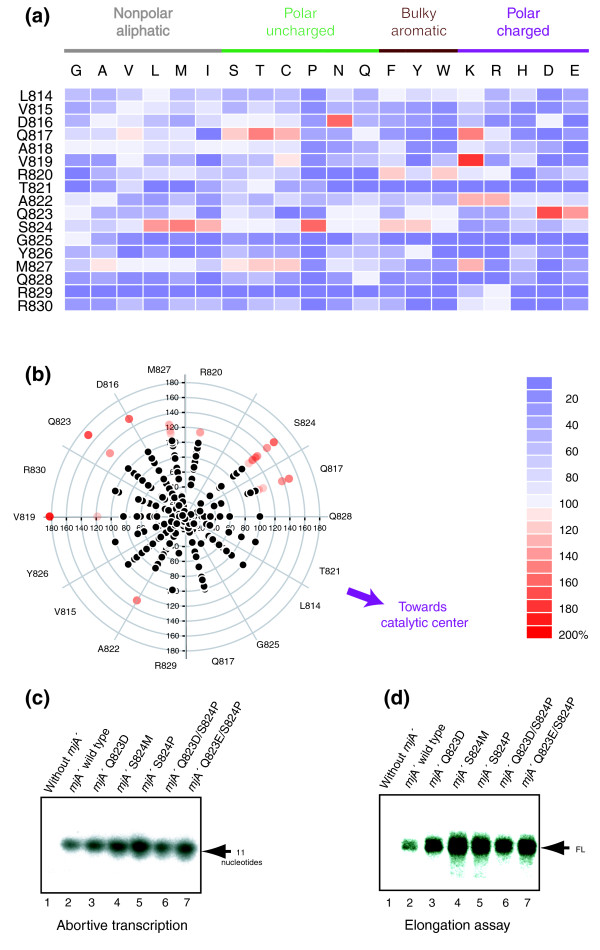
Activity assays of bridge helix mutants. **(a) **Graphical overview ('heat map') of the mutant activities from high-throughput non-specific transcription assays. The vertical axis shows the identity of the residues located along the *M. jannaschii *bridge helix, spanning the interval from L814 to R830 (inclusive). On the horizontal axis the amino acid substitutions for each of these positions is indicated. The specific transcriptional activities of the mutants are color-coded according to the scale shown lower right, ranging from inactive (dark blue, 0%) to superactive (dark red, 200%) relative to the wild-type activity (defined as 100%). The activity values for each substitution are based on a minimum of four independent assemblies and transcription assays (see Additional data files 1c, 2c, 3c, 4c, 5c, 6c, 7c, 8c, 9c, 10c, 11c, 12c, 13c, 14c, 15c, 16c, 17c for further details). Data for the *mj*A' G825 substitutions have been published previously [25] but are included here for completeness. **(b) **Polar plot ('helical wheel') of mutant activities reflecting the spatial arrangement of the residues relative to each other in the α-helical bridge helix. The activities of substitutions in individual residues (as labeled on the periphery) are plotted along the radius. Activities below the wild-type level (100%) are in black, whereas activities above that level are coded by their color and radial position. The figures along the 90°, 180°, 270° and 0/360° axes refer to percentage of wild-type activity. **(c) **Abortive transcription assays showing the incorporation of [α-^32^P]rUTP into abortive dinucleotide extension products on activated DNA during a 20-minute incubation period. **(d) **Multiple-round elongation transcription assays on a DNA-RNA scaffold. The position of the extension product is marked FL.

**Figure 3:**
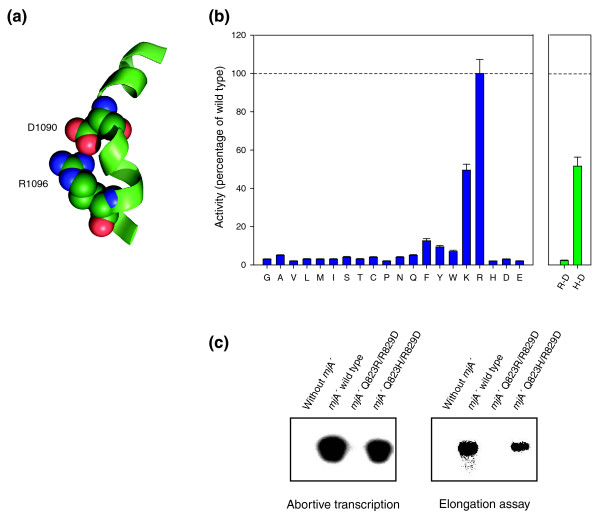
A functional interaction between the Q823 and R829 positions. **(a) **Model of the *T. thermophilus *bridge helix kink (PDB 1IW7). The interacting residues (β' D1090 and R1096) are shown as space-filling models and the surrounding helix in green in ribbon representation. Note that the flipped-out D1090 residue juxtaposes its side chain opposite R1096. The resulting contact stabilizes the kinked α helix. **(b) **High-throughput transcription assay results of *mj*A' R829X substitutions. The results are shown relative to wild-type activity (100%; dashed line). Single substitution mutant results are shown in dark blue with the substituted residues shown along the *x*-axis positions; note that all substitutions, except K, result in a substantial drop of catalytic activity. The results of two double mutant constructs, Q823R/R829D (R-D) and Q823H/R829D (H-D), are shown on the same scale as a separate graph with green bars. Error bars indicate standard deviation (*n *= 4). **(c) **Abortive and elongation transcription assay results of the double mutants. Q823R/R829D is inactive; Q823H/R829D has 49% (abortive assay) and 52% (elongation assay) of wild-type activity.

The bridge helix of *M. jannaschii *RNAP is located near the carboxyl terminus of the *mj*A' subunit and is clearly identifiable by its colinearity and high degree of sequence identity and/or similarity to bacterial and eukaryotic orthologs [25] (Figure 1b). The region chosen for the high-throughput mutagenesis approach is a stretch of 17 contiguous residues (*mj*A' L814 to *mj*A' R830 inclusive) that spans the active site (Figure 1a). We produced a library for each of these residues by creating targeted point mutations encoding all 19 possible single substitutions. The constructs encoding the mutants were expressed as recombinant subunits in *Escherichia coli*, purified and assembled in quadruplicate under identical conditions using the recently developed 'RNAP Factory' approach [25]. The parallel conditions for the growth, purification and *in vitro *assembly of a large number of mutant subunits (typically 96) provide a remarkable degree of consistency that allows the phenotypic effect of each mutation to be quantified robustly under defined *in vitro *conditions [25]. The resulting RNAP variants were initially screened using a high-throughput trichloroacetic acid (TCA) precipitation assay that measures the incorporation of [^32^P]rUTP into transcripts using nuclease-activated DNA as template. The bridge helix is part of the catalytic site of RNAPs, and these assays therefore provide a reliable and informative readout. We also tested a subset of mutants in separate dinucleotide extension assays that specifically measure abortive transcription events. These assays show that the effects of the various mutants on abortive transcription are comparable to the results obtained with the TCA-precipitation assays ([23] and LT and ROJW, unpublished results; see also Figures 2c,d and 3b).

The results of the transcription assays of 323 targeted mutants in the bridge helix reveal a broad spectrum of changes in the catalytic activities, varying from total loss of polymerase function to activities substantially exceeding the normal wild-type level (Figure 2a; Additional data files 1-17). The large variety of phenotypes observed is due to local alterations of side-chain chemistry (for example, gain or loss of charge and hydrophobic interactions) that either change the interactions with nearby molecular partners and/or affect intrinsic structural properties of the bridge helix. The site-directed mutations described here are targeted towards independently folded domains and are therefore unlikely to affect the conformation or stability of the overall RNAP structure (Additional data files 18 and 19; ROJW, unpublished observations).

As expected, many residues that seem to occupy critical positions in the previously published X-ray structures are particularly sensitive to change and cannot be substituted with any other amino acid without noticeable loss of activity. These include residues that interact with the rNTPs in the catalytic site (T821 in the single-letter amino acid code), or the DNA template strand entering the active site (T821, G825, Y826 and R829), thus confirming their essential roles. It is possible to deduce, for several positions in the bridge helix, the precise requirement for side-chain chemistry. This is easiest with residues for which most substitutions result in substantial loss of function. We have previously commented on the fact that for G825 the physical size of the side chain seems to be crucial because any additional atoms (other than the single hydrogen side chain of glycine) create a physical obstacle for the passing of the DNA template strand into the active site [25]. The phenotypes of T821 substitutions also reveal a high degree of sensitivity to alteration. Because of its location in the active site, the T821 side chain is placed in a unique position where, depending on the translocation state, the residue interacts either with the 3' OH end of the nascent transcript, or with the rNTP at the insertion site. Substitutions of T821 with alternative residues containing long, charged and/or bulky side chains lead to dramatic loss of function that is almost certainly caused by steric clashes and unfavorable intermolecular interactions.

It is similarly noticeable that the presence of a positively charged side chain in the R829 position seems to be absolutely critical (Figure 3b). Only R829K provides an active alternative, but even this rather conservative mutation incurs a substantial loss of function. At first glance, the location of R829 adjacent to the DNA template strand supports the idea that a positively charged residue may have a key role in this position, but there is evidence that this residue is also required for stabilizing an alternative conformation of the bridge helix [11] (see below for further details). In fact, we present evidence below that shows that, in particular double-mutant combinations, R829 can be replaced with a negatively charged residue (glutamic acid) and still support a reasonable level of catalytic activity.

Surprisingly, other residues that seem to be in spatially constrained positions in published X-ray structures can be replaced with a chemically diverse set of side chains without substantial loss of function. Inspection of X-ray structures of elongating RNAP complexes suggests that the A822 position would be unable to accommodate large side chains owing to steric clashes with the DNA template strand (see, for example, [5,6,11]). Substitutions of A822 with residues containing large, bulky and/or hydrophobic side chains (for example N, Q, F or Y) cause only a modest decrease of activity as compared with the wild-type enzyme. There is also a similarly unexpected tolerance to proline substitutions in certain bridge helix positions. The imino acid proline is fundamentally incapable of participating in α-helical conformations, restricts the conformational space of the residue at its amino-terminal side and disrupts the local hydrogen bonding pattern that stabilizes the secondary structure (see, for example, [26]). In many positions of the bridge helix, proline substitutions cause, as expected, a large loss of activity (summarized in Additional data file 20). In other positions (for example, T821P and A822P), a clearly detectable activity remains, and in one case (S824P) we found an astonishing increase of activity of the mutant in comparison with the wild type (Figure 2a; a more extensive interpretation of this phenotype is provided below). We deduce from the proline substitution phenotypes that there is no absolute requirement, at any stage of the nucleotide addition cycle, for the bridge helix to maintain the continuous α-helical conformation that has previously been observed very consistently in structural studies of elongating RNAPs (see, for example, [6-8]).

### Localized kinks in the bridge helix cause superactive catalysis

A third class of phenotype uncovered in the high-throughput screen is an unexpected large number of mutations (about 7% of the entire set) showing increased activity. We will refer to this phenomenon as 'superactivity' because it exceeds the normal wild-type level. The substitutions causing the catalytic enhancement are predominantly clustered in the D816, Q817, V819, Q823 and S824 positions. In addition, certain substitutions of R820, A822 and M827 result in more moderately increased levels of activity. A helical wheel projection shows that the side chains of D816, Q817, V819, Q823, S824 and M827 point away from the RNAP catalytic center (Figure 2b). This leads us to conclude that superactivity is not caused by the mutated side chains stimulating events in the active site directly; the observed phenotypes must instead be due to conformational changes in the structure of the bridge helix itself, and/or to an altered interaction pattern of the bridge helix with the adjacent trigger loop domain.

Two of the residues that can be mutated to superactivity (Q823 and S824) are orthologs of *Thermus thermophilus *(*tth*) subunit β' residues D1090 and S1091. In certain bacterial RNAP structures the two residues are present in a flipped-out configuration that disrupts the local hydrogen-bonding pattern of the α-helical structure [10-12]. These studies [10-12] have shown that *tth*β' D1090 (the ortholog of *mj*A' Q823) stabilizes the kinked conformation of the bridge helix through specific hydrogen bonding with a nearby invariant residue, *tth*β' R1096 [11] (Figures 1d and 3a). Such arginine-aspartate contacts are known to be of unusual strength, highly directional and, thus, particularly suitable for stabilizing intramolecular interactions [27]; there is also evidence that they can act as switches to stabilize alternative protein conformations ('ionic locks'; see, for example, [28]). It therefore seems that the *mj*A' Q823D substitution is capable of mimicking the aspartate-arginine stabilization pattern that is responsible for the kinked conformation of bacterial bridge helices. The enzyme containing Q823D is substantially more active than the wild type, suggesting that the kinked bridge helix represents a conformation that is highly favorable for the nucleotide addition cycle.

This interpretation of the Q823D phenotype receives further support from the most unusual mutant revealed in our screen. The superactive S824P substitution is also predicted to cause a kinked bridge helix conformation. When present in an α helix, proline residues distort the helical structure by consistently introducing a highly localized and permanent kink of about 26° [29]. Our results show that the placement of proline residues in the bridge helix sequence needs to be very precise to achieve this effect because proline substitutions in most other positions cause substantial, or even total, loss of activity (Figure 2a; Additional data file 20).

Increased levels of transcription can be the result of decreased abortive transcription rates favoring promoter clearance [30]. Dinucleotide extension assays confirmed, however, that the increased catalytic activities of the superactive mutants were reflected by comparable increases in abortive transcription. Under these conditions the RNAPs harboring Q823D and S824P have activities of about 135% and about 210%, respectively, relative to the wild-type enzyme (Figure 2c). The results show that the extent of kinking of the bridge helix predicted to be induced by Q823D and S824P does not seem to interfere in any way with the proposed template scrunching mechanism [31,32]. In addition, we investigated the elongation properties of the mutant RNAPs using factor-independent nucleic acid scaffolds under conditions allowing repeated initiation [23,33]. The results are directly comparable to the activities shown in the abortive transcription assays (Figure 2d). It is therefore clear that the superactive phenotypes are consistently observed in a variety of transcription assays. In comparison with the wild-type enzyme, superactive mutants assemble with equal efficiency, show identical chromatographic elution patterns and the same degree of thermostability (Additional data files 18 and 19; ROJW, unpublished data). The increased production of transcripts is thus solely a consequence of the enhanced catalytic activity, and it demonstrates that mutations in the bridge helix modulate the active site in a direct and rate-determining manner. In preliminary studies we tested the wild-type enzyme and RNAPs containing the superactive bridge helix substitutions for misincorporation of dTTP in non-specific, abortive and elongation assays and have so far found no detectable loss of selectivity in rNTP incorporation (data not shown).

The conclusions from two independently acquired pieces of evidence thus converge on the same explanation: the superactive mutations Q823D and S824P are capable of creating and/or stabilizing a localized kink in a precisely defined region of the bridge helix. Because these mutations seem to achieve a similar result using different structural principles, we investigated the effects of double mutant combinations. Constructs containing Q823 and S824 substitutions in combinatorial configurations were used to create Q823D/S824M, Q823D/S824P, Q823E/S824M and Q823E/S824P double mutants. The double mutants showed similar levels of elevated activity as Q823D and S824P on their own, demonstrating that no further gain of function is achievable (Figure 2c,d; Additional data file 19).

A final piece of evidence in support of an interaction between Q823 and R829 comes from a stringent test using another set of double mutants. Taking into account the stabilizing interactions between *tth*β' D1090 and *tth*β' R1096 [8] (Figure 3a), we wondered whether it would be feasible to recreate this interaction by switching the positions of these residues. Although a Q823R/R829D double substitution was inactive, another, Q823H/R829D, had 47–50% of wild-type activity (Figure 3b,c). We consider this result to be remarkable, taking into account the fact that R829D is completely inactive (like any other substitution in that position except, to a certain extent, lysine; Figure 3b). The presence of a histidine residue in position 823 thus rescues, to a significant extent, the R829D phenotype in a manner consistent with the predicted local interaction between these two positions during bridge helix kinking.

Each of the superactive point mutants is capable of causing the phenotype to the fullest possible extent on its own, and the absence of additive or synergistic effects is compatible with the view that the mutants kink the bridge helix in a similar manner. Structural evidence for bridge helix kinking was previously observed only in bacterial RNAPs [10-12]. The data presented here reveal for the first time a common link between the hitherto distinct bridge helix conformations in bacterial and archaeal RNAPs. Given that archaeal bridge helices are more akin to their eukaryotic counterparts than are the bacterial bridge helices, a plausible implication of this argument is that localized bridge helix kinking forms part of the normal RNAP nucleotide addition cycle across the entire evolutionary range.

### The trigger loop base helices are structurally delicately balanced

**Figure 4:**
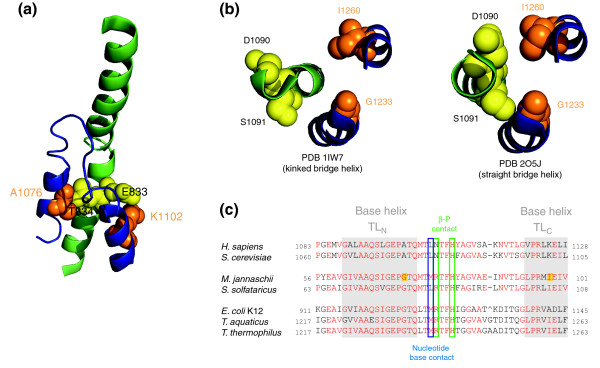
Structure of the trigger loop and its interface to the bridge helix. **(a) **Overview of the spatial relationship between the trigger loop (blue ribbon representation) and the bridge helix (green ribbon representation) based on a yeast RNAPII X-ray structure containing a folded trigger loop structure [7] (PDB 2E2H). Bridge helix residues E833 and T834 (yellow) and trigger loop residues A1076 and K1102 (orange) are shown as space-filling models. **(b) **Spatial relationship of the trigger loop base helices with the bridge helix in the kinked (PDB 1IW7) and straight (PDB 2O5J) versions of *T. thermophilus *RNAP. Note how the flipping out of bridge helix residues D1090 and S1091 during kinking disrupts their proximity to trigger loop residues I1260 and G1233, respectively. **(c) **Sequence alignments of trigger loop sequences using the same criteria as for the bridge helix alignments shown in Figure 1b. TL-N and TL-C indicate the amino-terminal and carboxy-terminal trigger loop base helices, respectively.

The residues that can be mutated to superactivity are predominantly located at the 'back' of the bridge helix (facing away from the catalytic site). Inspection of the bacterial, archaeal and yeast elongating RNAP structures shows that the bridge helix residues orthologous to *mj*A' Q823 and S824 are close to the adjacent trigger loop [5-9]. Two short α helices form two pillar-like structures at the bases of the trigger loop and are connected by a flexible 'tip' region (Figures 1a and 4a). This tip region tends to be unstructured, but can also take up a variety of conformations in the presence of substrates or inhibitors [7,8,13]. A network of contacts between the tip and various parts of the rNTP is likely to promote catalysis in an as yet unknown manner, most likely through the precise positioning of the nucleotide substrate relative to the active site. In agreement with this model, a variety of mutations in the trigger loop has been shown to affect substrate usage and enzyme fidelity [17,21,34].

The spatial vicinity between the bridge helix residues and trigger loop base helix residues prompted us to investigate the possible significance of these contacts in more detail. Residues orthologous to *mj*A' Q823 touch a specific residue in the carboxy-terminal trigger loop base helix (abbreviated as TL_C _from here on) that corresponds to residue I98 of the RNAP *mj*A'' subunit. Conversely, residues orthologous to S824 touch another residue in the amino-terminal trigger loop base helix (TL_N_), which corresponds to *mj*A'' G72 (Figure 4c). Given the geometry of α helices (which imposes an angle of about 100° between adjacent amino acids), the bridge helix is thus capable of contacting both trigger loop base helices using only two successive residues. The contacts of Q823 and S824 with TL_C _and TL_N_, respectively, could constitute an important functional interface between the bridge helix and trigger loop. We therefore created two more libraries containing all possible substitutions in *mj*A'' G72 and *mj*A'' I98, respectively, to study the phenotypic effects.

**Figure 5:**
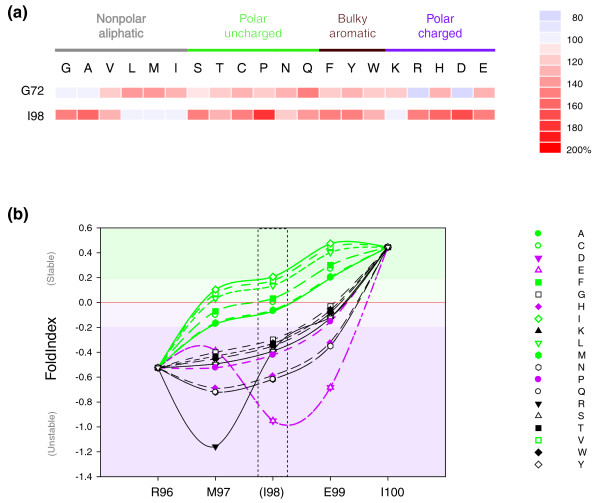
Activity assays of trigger loop mutants. **(a) **Graphical overview ('heat map') of the mutant activities from high-throughput non-specific transcription assays. The vertical axis shows the identity of the residues located along the *M. jannaschii *trigger loop. On the horizontal axis the amino acid substitutions for each of these positions is marked. The transcriptional activities of the mutants are color-coded according to the scale shown in the right relative to the wild-type activity (defined as 100%). The activity values for each substitution are based on a minimum of four independent assemblies/transcription assays. **(b) **Prediction of local stabilities of substitutions in the *mj*A'' I98 position. The 'FoldIndex' was calculated using a web-based program, FoldIndex [48] (window size = 3; step = 1) with *mj*A'' amino acid sequences containing substitutions with all 20 different amino acids in the I98 position. Areas within the graph with positive FoldIndex values (stable folding) and negative values (unstable folding) are indicated by a light green or magenta background, respectively. I98 seems to occupy a critical area between regions of low stability (R96) and high stability (E99 and I100). The identity of residue 98 (highlighted between the dashed lines) critically determines the shape of this transition; a small number of hydrophobic residues increases local stability (I>V>L>F>C>M>A; green symbols), whereas most other substitutions cause local destabilization (for example P, D or E; magenta). Certain substitutions may cause additional structural changes that cannot be accounted for by this prediction. The symbols for the various amino acids are shown on the right.

The results reveal a highly unusual pattern. Essentially none of the 19 alternative substitutions in either trigger loop base helix residue causes any substantial reduction in transcriptional activity as measured by the high-throughput transcription assays (Figure 5a). In fact, the majority of substitutions cause superactivity that reaches (for example, in the case of I98P) a level that is indistinguishable from the effects seen with some of the substitutions in the neighboring bridge helix. The results also reveal that, in the G72 (TL_N_) position, only glycine or alanine, and in the I98 (TL_C_) position, only a very select number of other substitutions (I, V, L, M or K), are capable of providing the relatively low levels of activity (rather than high levels, as might be expected) that are apparently required for wild-type function. Side-chain identity therefore has only a minor role for these positions (note, for example, that, in the case of *mj*A'' I98, very similar activities were observed using A, G, S, T, C, F, Y and R substitutions, residues with radically different chemical properties), suggesting that one of the major factors influencing the function of the trigger loop base helices TL_N _and TL_C _may be local stability, rather than specific side-chain chemistry. This interpretation is supported by a good match of our experimental data with results from a bioinformatic analysis aimed at detecting intrinsically unfolded sequences from local hydrophobicity and net charge densities [35,36] (Additional data file 21).

This investigation reveals that the *mj*A'' I98 (TL_C_) position is intrinsically weakly stable and becomes easily disordered when substituted by almost all residues identified in the trigger loop mutagenesis screen that convert the RNAP to superactivity (Figure 5b). The presence of a highly conserved G-X-P hinge motif [37] nearby may be important in this conformational switch. A similar study classifies the region surrounding G72 as unstable (Additional data file 21). We therefore propose that the trigger loop base helices TL_N _and TL_C _are finely poised at the edge of structural stability. Even minor variations (such as the replacement of either *mj*A'' G72 or *mj*A'' I98 with other residues by site-directed mutagenesis) cause a substantial loss of local stability by altering the local net charge/hydrophobicity ratio. In bacterial RNAPs, TL_N _and TL_C _are capable of adopting alternative conformations, possibly in response to structural changes in the hinge region of the bridge helix [12]. Similarly, in yeast RNAPII the scRpb1 E1103G substitution (corresponding to *mj*A''E99, that is, immediately carboxy-terminal to the *mj*A'' I98 in TL_C_; Figure 4) shows increased catalytic activity [21,34,38]. These results provide a plausible explanation for the superactive phenotypes observed with certain substitutions in bridge helix residues. Some of the mutations in Q823 and S824 destabilize TL_N _and TL_C _by kinking this part of the bridge helix away from the trigger loop base helices, thus causing conformational changes in the trigger loop that increase the catalytic activity (Figure 4b). We also imagine that similar events are likely to occur in the superactive mutations located in more amino-terminal regions of the bridge helix, such as D816, Q817 and V819. The precise contact points between the bridge helix and trigger loop in these regions are, however, not as clearly definable because different trigger loop orientations have been observed in RNAP crystal structures [7,8,12].

Finally we created various recombinant RNAPs containing combinations of superactive bridge helix and superactive trigger loop mutants, such as *mj*A' S824P/*mj*A'' I98P. Just as previously observed with the bridge helix double mutants, no further increase in superactivity was detected (data not shown). Single point mutants in either the bridge helix or the trigger loop are therefore sufficient to induce the full superactivity phenotype. The lack of additivity or synergism suggests that each mutant affects the same process in a functionally overlapping and mutually independent manner.

## Discussion

Although the chemical aspects of the catalytic functions of nucleic acid polymerases are well established [39], there is still a considerable amount of uncertainty concerning the mechanical aspects that link these catalytic steps to movement of the nucleic acid substrates through the active site.

RNAPs are powerful nanomechanical devices that carry out transcription at considerable speed [40] and exert forces that exceed cytoskeletal motors [15,41].

In this study we describe the most extensive example of a high-throughput structure-function analysis so far that relies on neither genetic screens to isolate mutants nor the use of site-directed mutagenesis to test a preconceived model. Instead, we implemented a new experimental approach that is designed to sample systematically a substantial area of protein structure-function space. The collection of such large datasets is especially important for complex macro-molecular machines that undergo substantial conformational changes at different stages of the reaction cycle that might not be obvious from the small numbers of 'canonical' high-resolution structures available [42]. Many of the most informative mutants discovered in the screen would not have been designed using prior knowledge, either because there would have been no rational reason to do so (for example, V818K), or because the likelihood of obtaining useful insights would have been regarded as too low to justify the experimental effort (for example, S824P).

The results shed new light on the mutual relationship between the bridge helix and trigger loop. Specifically, we show that the molecular contacts made between the bridge helix and trigger loop are influenced by the conformations of the two domains (localized kinking of the bridge helix and stability of the trigger loop base helices). The preponderance of the straight bridge helix conformation in the majority of available structures has resulted in the kinked versions often being dismissed as artifacts or 'off-pathway' conformations. Results shown here prove that kinked bridge helix conformations are indeed compatible with catalytic function and even capable of supporting rates that exceed wild-type activity by a considerable measure. We suggest that bridge helix kinking is a normal (although possibly short-lived) intermediate conformational state of RNAP and that the enhanced catalytic rates observed in some of the mutants are the result of a bias towards this state. Such an interpretation is in general agreement with the original models proposed for RNAP function [5,6,8,14,17], rather than more recent trigger loop-centric hypotheses [3,18].

**Figure 6:**
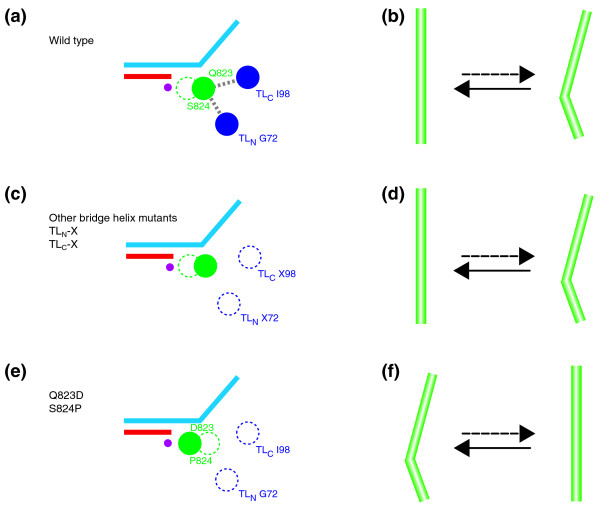
Models of bridge helix and trigger loop mutant effects. Summary of the predicted positions of the bridge helix (green) and trigger loop base helices (dark blue). **(a, c, e) **The helices are shown as a schematic cross-section (top view, similar to Figure 1c) to indicate their locations relative to each other. The DNA template strand is shown in light blue and the newly synthesized transcript in red. The amino acids specifically labeled refer to *M. jannaschii *positions in the A' (Q823, S824) and A'' (I98 and G72) subunits, respectively. The position of the catalytic site is represented by the 'Metal A' ion as a magenta dot. **(b, d, f) **Schematic side views of the bridge helix (similar to Figures 1a, d) to illustrate the proposed equilibrium distribution between straight and kinked conformations in the wild-type and mutant enzymes. **(a, b) **In the wild-type, the bridge helix and trigger loop base helices are typically in close contact (indicated by the gray dotted lines in (a)) and the bridge helix is predominantly found in the straight conformation (b). The contacts between the bridge helix and trigger loop stabilize the conformation of the trigger loop base helices. **(c, d) **In some of the bridge helix mutants, and nearly all the trigger loop mutants described here (TL_N_-X72 and TL_C_-X98), contacts between bridge helix and the trigger loop are diminished, although the bridge helix conformation is unaffected. **(e, f) **In certain bridge helix mutants (especially Q823D and S824P), the kinked bridge helix is mainly in the 'forward' position and is therefore not capable of maintaining effective contacts with the trigger loop base helices.

It is nevertheless clear that not all observed superactive phenotypes are exclusively caused by conformational changes in the bridge helix. Independent mutations in the trigger loop base helices and other point mutants in the bridge helix that are likely to affect the bridge helix/trigger loop interface also cause similar increases in the catalytic activity. We therefore propose a model that explains these apparently separate phenotypic classes as the perturbation of a common mechanism in which both domains participate (Figure 6). According to this scheme, the trigger loop base helices are delicately balanced on the verge of instability and require bridge helix residues nearby in order to form a stable three-helix bundle (Figure 6a,b; Additional data file 21). If these interactions are disrupted by mutations (Figure 6c,d), or through preferential bridge helix kinking towards the active site (Figure 6e,f), the trigger loop base becomes more mobile. This increased mobility of the trigger loop is, in turn, responsible for the superactive phenotype.

The more amino-terminal bridge helix mutants (for example, V819K, Q817T and D816N) probably act in a similar manner by weakening trigger loop contacts in the region closer to the active site, but they may also exert their effects more indirectly through as yet undefined local changes in bridge helix folding and stability (Additional data file 21). We therefore propose that the flexibility of the trigger loop is directly influenced by the conformation of the bridge helix. Any loss of this bridge-helix-induced constraint on the trigger loop allows the trigger loop to remain longer in a conformation favorable to catalysis and thus give rise to the superactive phenotypes observed.

We further suggest that under normal conditions a periodic transition between the straight and kinked version of the bridge helix allows the trigger loop to take up temporarily a conformation that is highly favorable for the execution of the catalytic reaction before it becomes restrained again through contacts along the bridge helix/trigger loop interface. Normal wild-type activity may therefore be the consequence of temporary bursts of catalytic activity, which are more prolonged in the superactive mutants described here. We currently do not understand how the trigger loop interacts with the catalytic site to promote phosphodiester bond formation, but it is feasible that a kinked bridge helix conformation stabilizes the post-translocation state of the DNA-RNA hybrid and thus provides the trigger loop tip domain with an increased opportunity to sequester an incoming rNTP in a steric manner most favorable for phosphodiester bond formation [7,8,18,34]. A recent report also suggests that bridge helix kinks could facilitate the conformation of the pre-insertion position of the DNA template strand; such a phenomenon could also explain, at least in part, the increased level of activity in some of the bridge helix mutants described above [13].

## Conclusion

The high-throughput mutagenesis data show that the bridge helix of *M. jannaschii *RNAP subunit *mj*A', in combination with the trigger loop, has a major impact on the catalytic activity of RNAP. The extent of this effect is striking: single point mutants in these domains cause functional effects that range from complete abolition of enzyme function to a near-doubling of the catalytic rate without any additional changes anywhere else among the up to 3,500 other amino acids that make up a complete multisubunit RNAP. Although our results are currently restricted to an archaeal *in vitro *system, it is very likely that many of the features described here are universal, and we expect that it will be possible to create bridge helix mutants with similar properties in other well-studied organisms, such as *E. coli *and *Saccharomyces cerevisiae*. Furthermore, the variations displayed by the superactive mutations in the bridge helix/trigger loop domains prove that the catalytic rate of RNAPs is intrinsically subject to variation and is, at least under *in vitro *conditions, not programmed to its maximum level. Interactions with regulatory proteins (especially elongation and anti-termination factors) can modulate the active site by stabilizing different conformational states (Figure 6), and evolutionary changes in the bridge helix and trigger loop sequences can 'fine tune' the catalytic capacity of cellular enzymes for an optimum rate in the long term.

It has previously been suggested that in prokaryotes the RNAP elongation rate may be optimized for allowing RNA folding or co-translation and in eukaryotes for post-transcriptional processing of primary transcripts [43-45]. Inspection of the amino acids present in certain rate-determining bridge helix positions shows an intriguing degree of species-dependent variation (Figures 1b and 4c; Additional data files 1-17), which suggests that such adaptations are indeed used during evolution to continuously coordinate the functional properties of RNAPs with other processes involved in gene expression.

## Materials and methods

### Mutagenesis

The generation of site-directed mutants using oligonucleotides with randomized codon positions (*mj*A'A818, V819, R820, T821, A822, Q823, S824, G825, Y826, M827, Q828, R829 and R830) was carried out as described in Nottebaum *et al*. [25]. Briefly, the segment of bacterial expression vectors encoding the bridge helix domain was replaced with double-stranded oligonucleotides containing randomized positions corresponding to the codon targeted for muta-genesis. Constructs containing the desired amino acid substitutions were selected from a collection of randomly picked clones after sequencing. For residues *mj*A' L814, V815, D816, Q817 and *mj*A'' G72 and I98, sequential permutation libraries were constructed from custom synthetic libraries purchased from GeneArt (Regensburg, Germany). Each mutant construct described in this study was validated at least once by DNA sequencing to confirm the presence of the expected point mutation and the integrity of the restriction enzyme sites used for the subcloning procedures.

### Large-scale archiving and growth of mutants

The expression plasmids were stored as arrayed frozen bacterial expression strain stocks in two-dimensionally barcoded tubes at -80°C in the presence of 5% dimethyl sulfoxide as anti-freezing agent. For each mutagenized amino acid position, all substitutions were arranged in a standardized pattern with multiple wild-type and negative controls. For recombinant protein production, four 24-deepwell plates containing 1.5 ml per well of autoinduction medium (Novagen) were robotically inoculated from these frozen stocks and grown with shaking at 37°C for 16 h before further processing.

### High-throughput subunit purification

The purification of mutant and wild-type *mj*A' subunits was carried out robotically in sets of 96 as described previously [25]. Briefly, aliquots of the induced cultures were lyzed in deepwell plates using chemical and enzymatic agents [FastBreak (Promega) and Lysonase (Novagen), respectively]. The recombinant *mj*A' subunits were then purified from the lysates as inclusion bodies and solubilized in the presence of near-saturating (8.3 M) urea. The protein concentrations of the subunit preparations were automatically monitored with the Bicinchonic Acid assay (Sigma). A similar procedure was adapted for the purification of recombinant *mj*A'' subunits by including 10% isopropanol in the wash buffer to reduce the solubility of the recombinant proteins during the inclusion body purification step. This procedure typically yielded about 250 μg of purified recombinant subunits from 900 μl expression culture with a standard deviation in the concentrations of individual subunit preparations of less than ± 10% (the presence of the point mutations had no discernible effect on the growth of expression cultures or on the yield and solubility of the recombinant proteins).

### High-throughput *in vitro *assembly of RNAPs

The assembly procedure was carried out robotically as previously described [25]. Small-scale *in vitro *assembly reactions (final volume 100 μl) were robotically prepared by combining aliquots of the *mj*A' mutant subunits with a 'Master Mix (-A')' containing an empirically optimized mixture of the other RNAP subunits in 6 M urea (the subunits present in the Master Mix are rate-limiting in the assembly reactions; variations in the mutant *mj*A' subunit concentrations thus do not influence the final yield of assembled RNAP). The assembly mixtures were then transferred to a 96-well microdialysis device (Spectrum Laboratories). The RNAPs were automatically assembled by gradually lowering the urea concentration in the dialysis chamber from 6 M to urea-free over a period of 16 h using a robotically controlled pump. For chromatographic analyses (Additional data file 18), 350 μl assembly mixes were separated on a Superose-12 10/300 High Performance column (GE Healthcare) on a BioLogic Duoflow system (Bio-Rad) at a flow rate of 0.25 ml/minute in urea-free assembly buffer [25]. The eluate was monitored with a Quad-Tech detector (Bio-Rad) and fractions collected (350 μl each) were analyzed for RNAP activity using the automated TCA precipitation assay described below.

### Transcription assays

TCA precipitation assays measuring the incorporation of [α-^32^P]rUTP into TCA-insoluble products were carried out as previously described [22,23]. For the robotic implementation of this assay [25], aliquots of the assay mixtures were incubated for 45 minutes at 70°C in thin-wall PCR plates. The radiolabeled transcripts were then precipitated by the addition of ice-cold TCA solution. After incubation for 30 minutes at 1°C, the mixture was robotically pipetted onto a 96-GF/F glass fiber filter plate (Whatman) on a robotic vacuum platform. Unincorporated [α-^32^P]rUTP was filtered to waste and the labeled RNA retained on the filter surface was washed seven times with further aliquots of ice-cold TCA. After additional washes with 2-propanol and vacuum drying, the amount of incorporated [α-^32^P]rUTP was quantified with a microplate counter (TopCount NXT, Packard) in the presence of scintillant (MicroScint-O; Perkin-Elmer).

The dinucleotide extension (abortive) assays were performed manually as previously described [23]. RNAPs were incubated at 70°C for 30 minutes with activated DNA (Sigma Type XV), CpG dinucleotide and [α-^32^P]rUTP. The extension products were separated from unincorporated label on 20% acrylamide gels, visualized by phosphoimaging (Fuji) and quantified (AIDA image analyzer; Raytest). The multiple-round elongation assays used a promoter-independent nucleic acid scaffold (EC3) that mimics an elongation transcription complex [46]. This scaffold contains a nine-nucleotide RNA pre-hybridized to the template strand, which is extended into a 71-nucleotide run-off transcript by RNAP (in the absence of basal transcription factors). Elongation reactions were preincubated for 20 minutes at 60°C in 20 μl TB (50 mM Tris-HCl, pH 7.5, 75 mM KCl, 2.5 mM MgCl_2_, 10 mM dithiothreitol, 8 pmol annealed ECR3 scaffold [46] and about 100 ng RNAP) before transcription (20 minutes at 60°C) was initiated by the addition of NTPs [500 μM rATP, 500 μM rCTP, 500 μM rGTP, 10 μM rUTP and 0.15 MBq [α-^32^P]rUTP (110 TBq/mmol)]. The analysis and quantification of the extension products was carried out as described above for the dinucleotide extension assay. For all transcription assays the incubation periods were in the linear response range.

## Additional data files

The following additional data are available. Additional data file 1 shows the structure, evolution and function of *mj*A' L814. Additional data file 2 shows the structure, evolution and function of *mj*A' V815. Additional data file 3 shows the structure, evolution and function of *mj*A' D816. Additional data file 4 shows the structure, evolution and function of *mj*A' Q817. Additional data file 5 shows the structure, evolution and function of *mj*A' A818. Additional data file 6 shows the structure, evolution and function of *mj*A' V819. Additional data file 7 shows the structure, evolution and function of *mj*A' R820. Additional data file 8 shows the structure, evolution and function of *mj*A' T821. Additional data file 9 shows the structure, evolution and function of *mj*A' A822. Additional data file 10 shows the structure, evolution and function of *mj*A' Q823. Additional data file 11 shows the structure, evolution and function of *mj*A' S824. Additional data file 12 shows the structure, evolution and function of *mj*A' G825. Additional data file 13 shows the structure, evolution and function of *mj*A' Y826. Additional data file 14 shows the structure, evolution and function of *mj*A' M827. Additional data file 15 shows the structure, evolution and function of *mj*A' Q828. Additional data file 16 shows the structure, evolution and function of *mj*A' R829. Additional data file 17 shows the structure, evolution and function of *mj*A' R830. Additional data file 18 shows the chromatographic elution profiles of wild-type and mutant mjRNAPs. Additional data file 19 shows the activities of wild-type and mutant mjRNAPs at limiting and saturating template DNA concentrations. Additional data file 20 shows the functional consequences of proline substitutions in different bridge helix positions. Additional data file 21 contains the bioinformatic analysis of intrinsic folding properties of bridge helices and trigger loops.

## Supplementary Material

Additional file 1 Structure, evolution and function of *mj*A' L814. Click here for file

Additional file 2 Structure, evolution and function of *mj*A' V815. Click here for file

Additional file 3 Structure, evolution and function of *mj*A' D816. Click here for file

Additional file 4 Structure, evolution and function of *mj*A' Q817. Click here for file

Additional file 5 Structure, evolution and function of *mj*A' A818. Click here for file

Additional file 6 Structure, evolution and function of *mj*A' V819. Click here for file

Additional file 7 Structure, evolution and function of *mj*A' R820. Click here for file

Additional file 8 Structure, evolution and function of *mj*A' T821. Click here for file

Additional file 9 Structure, evolution and function of *mj*A' A822. Click here for file

Additional file 10 Structure, evolution and function of *mj*A' Q823. Click here for file

Additional file 11 Structure, evolution and function of *mj*A' S824. Click here for file

Additional file 12 Structure, evolution and function of *mj*A' G825. Click here for file

Additional file 13 Structure, evolution and function of *mj*A' Y826. Click here for file

Additional file 14 Structure, evolution and function of *mj*A' M827. Click here for file

Additional file 15 Structure, evolution and function of *mj*A' Q828. Click here for file

Additional file 16 Structure, evolution and function of *mj*A' R829. Click here for file

Additional file 17 Structure, evolution and function of *mj*A' R830. Click here for file

Additional file 18 Chromatographic elution profiles of wild-type and mutant *mj*RNAPs. Click here for file

Additional file 19 Activities of wild-type and mutant *mj*RNAPs at limiting and saturating template DNA concentrations. Click here for file

Additional file 20 Functional consequences of proline substitutions in different bridge helix positions. Click here for file

Additional file 21 Bioinformatic analysis of intrinsic folding properties of bridge helices and trigger loops. Click here for file
