# Supplementary material for: Bridge helix and trigger loop perturbations generate superactive RNA polymerases
Source: J Biol. 2008 Dec 2;7(10):40. doi: 10.1186/jbiol98 (PMC2776397; doi:10.1186/jbiol98)
Supplement: Additional file 14 — Structure, evolution and function of mjA' M827. [file jbiol98-S14.pdf]

Identity and location of the mutated bridge helix residue (from *M. jannaschii*): *mj*A' M827

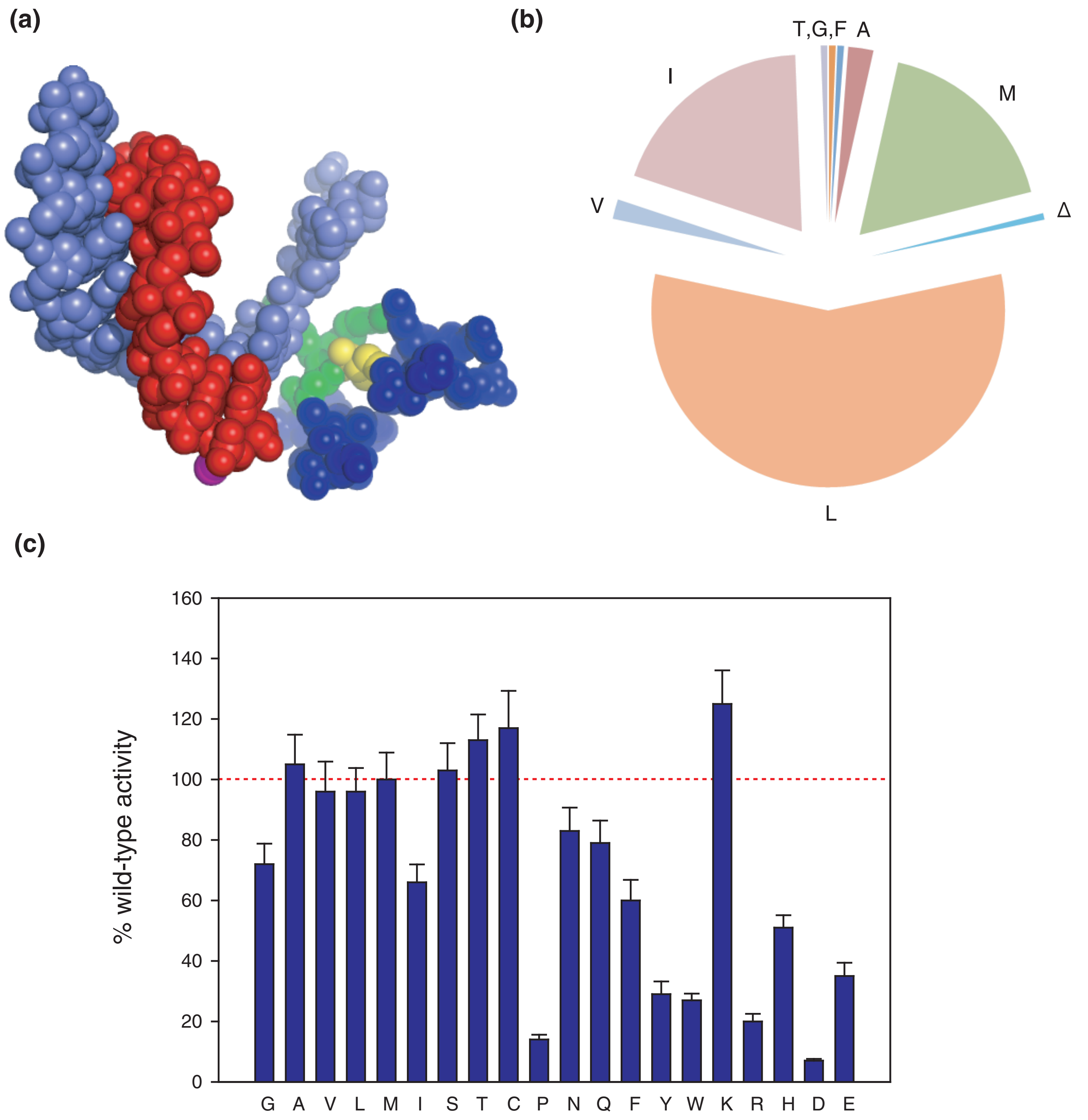

Additional data file 14. Structure, evolution and function of *mj*A' M827

(a) Spatial position of the mutated residue (yellow) relative to the DNA template strand (light blue), nascent transcript (red), bridge helix (green) and the trigger loop base helices (dark blue). The figure is based on the structure of yeast RNAPII (PDB code 1I6H).
(b) Pie chart representing the type and proportion of amino acid residues found in the orthologous position across the evolutionary range. Amino acids are identified by the single letter code.
The data are based on an alignment of 171 unique bridge helix sequences (containing archaeal, bacterial and eukaryotic entries) generated with FRPred using the *M. jannaschii* bridge helix sequence as probe [47]. The Δ symbol indicates an absence of the residue in one or more bridge helices.
(c) Results of [α-^{32}P]rUTP incorporation into TCA-precipitable counts on nuclease-activated DNA. The specific activities of substitutions with different residues (as identified along the horizontal axis) are shown relative to the activity of the enzyme containing the wild-type bridge helix sequence (defined as 100%; also marked by a horizontal red dotted line). The error bars indicate standard deviation from at least four independent recombinant subunit preparations and *in vitro* assemblies.
