# Supplementary material for: Bridge helix and trigger loop perturbations generate superactive RNA polymerases
Source: J Biol. 2008 Dec 2;7(10):40. doi: 10.1186/jbiol98 (PMC2776397; doi:10.1186/jbiol98)
Supplement: Additional file 18 — Chromatographic elution profiles of wild-type and mutant mjRNAPs. [file jbiol98-S18.pdf]

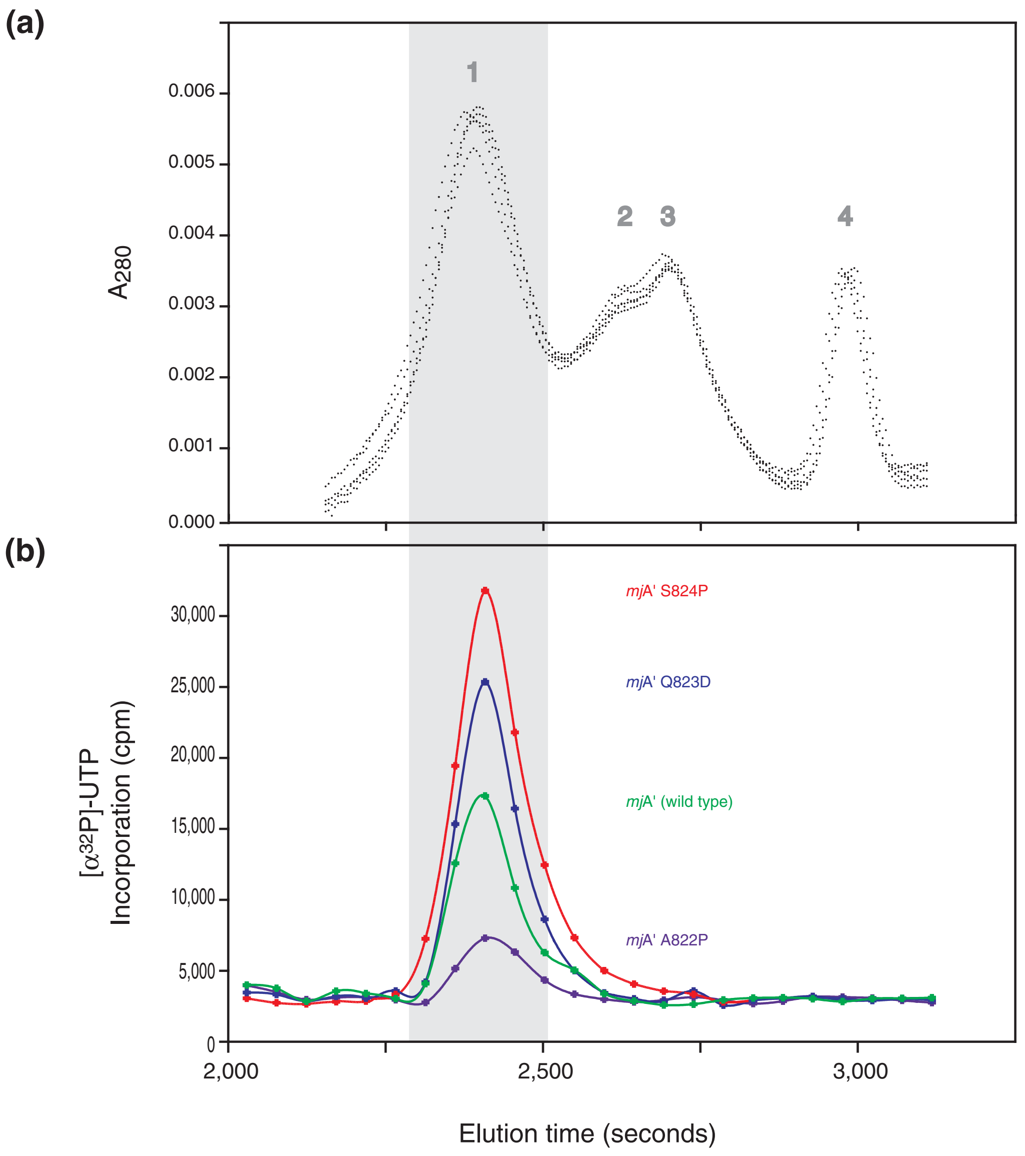

Additional data file 18. Chromatographic elution profiles of wildtype and mutant *mj*RNAPs

(a) Wild-type *mj*RNAP and three mutant variants (*mj*A A822P, *mj*A Q823D and *mj*A S824P) have essentially identical elution profiles during size-exclusion chromatography on a Superose-12 column. The dots represent individual readings of the detector measuring absorption at 280 nm. Peak 1 identifies fully assembled and catalytically active RNAP (this peak is absent in assembly reactions lacking the A´ subunit). Other nearby peaks (2, 3 and 4) represent partially assembled complexes that are catalytically inactive.
(b) TCA transcription assay results of the fractions corresponding to the elution profile shown in (a). The activities correlating with peak 1 show the same variations as the activities detected in the TCA assays with unfractionated assemblies.
