# Supplementary material for: Bridge helix and trigger loop perturbations generate superactive RNA polymerases
Source: J Biol. 2008 Dec 2;7(10):40. doi: 10.1186/jbiol98 (PMC2776397; doi:10.1186/jbiol98)
Supplement: Additional file 19 — Activities of wild-type and mutant mjRNAPs at limiting and saturating template DNA concentrations. [file jbiol98-S19.pdf]

(a)

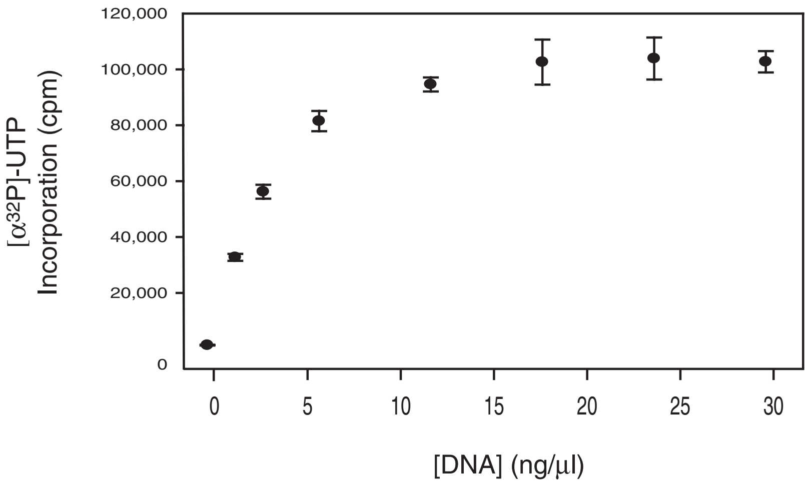

(b)

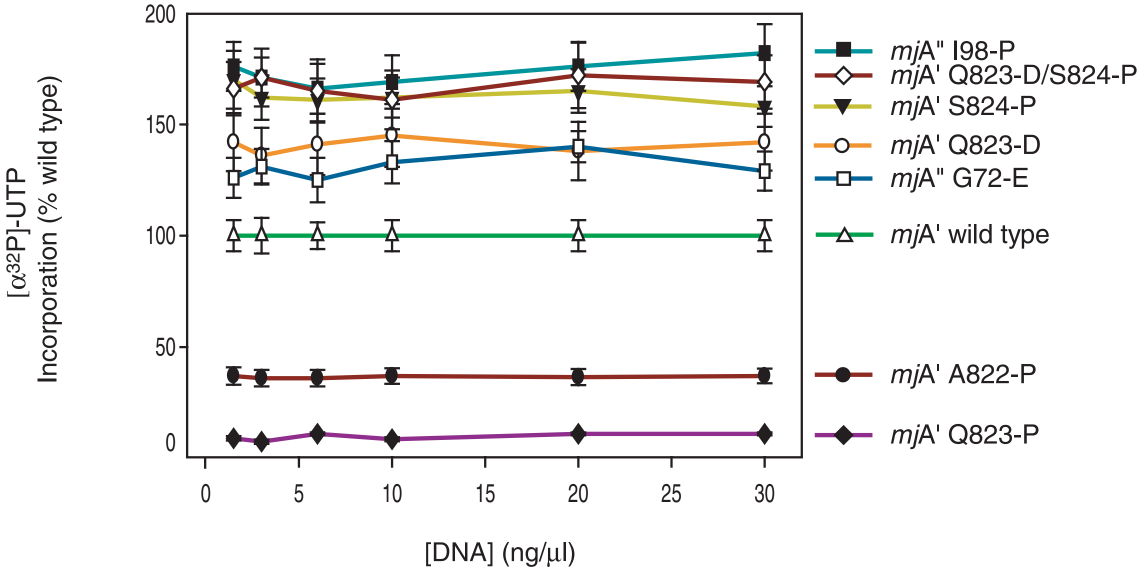

Additional Data File 19. Activities of wild-type and mutant *mj*RNAPs at limiting and saturating template DNA concentrations

(a) Transcriptional activity of wild-type *mj*RNAP in TCA-precipitation assays carried out in the presence of different template DNA concentrations at a fixed enzyme concentration. Template DNA concentrations below about 15 ng/μl become rate-limiting during the 30 minute incubation period.

(b) Results of TCA-precipitation assays carried out at limiting (1.5 ng/μl, 3 ng/μl, 6 ng/μl and 10 ng/μl) and saturating (20 ng/μl and 30 ng/μl) template DNA concentrations in the presence of constant amounts of RNAPs containing wild-type or mutant *mj*A' subunits. The activities of the various mutants (identified by the color code on the right) remain constant relative to the wild-type enzyme under subsaturating and saturating DNA concentrations, showing that the results reflect genuine differences in specific activity. The thermostabilities of the mutant enzymes are identical to that of wild-type *mj*RNAP (half-life of 57 ± 5 minutes at 70°C; ROJW, unpublished observations).
