# Supplementary material for: Bridge helix and trigger loop perturbations generate superactive RNA polymerases
Source: J Biol. 2008 Dec 2;7(10):40. doi: 10.1186/jbiol98 (PMC2776397; doi:10.1186/jbiol98)
Supplement: Additional file 20 — Functional consequences of proline substitutions in different bridge helix positions. [file jbiol98-S20.pdf]

Bridge Helix: Proline substitutions

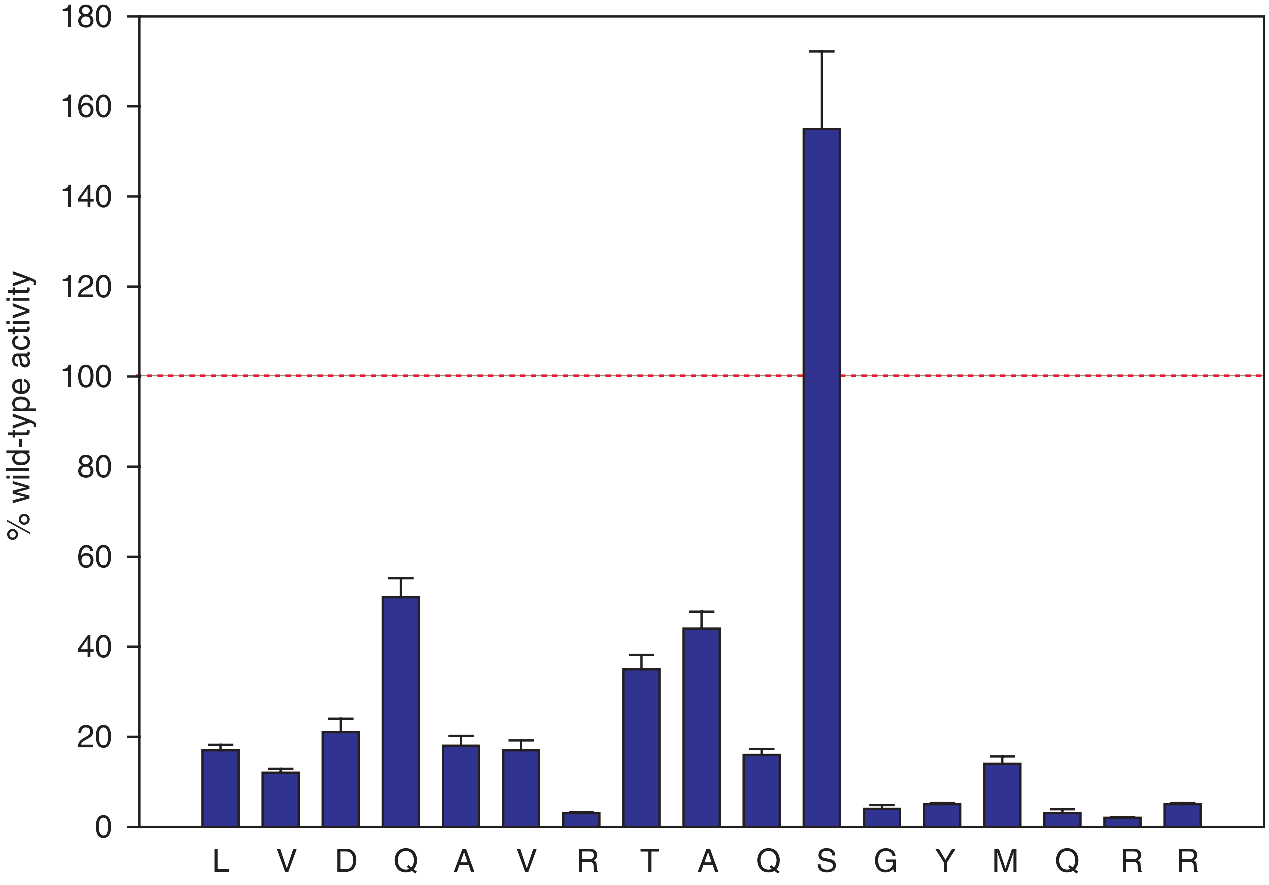

Additional data file 20. Functional consequences of proline substitutions in different bridge helix positions
Compilation of the proline substitution data extracted from Additional data files 1c-17c.

The sequence of the *mj*A' bridge helix is shown on the horizontal axis (L814 to R830) and the activity of the proline replacement in that position is plotted above. The specific activities of proline substitutions are shown relative to the activity of the enzyme containing the wild-type bridge helix sequence (defined as 100%; also marked by a horizontal red dotted line). The error bars indicate standard deviation from at least four independent recombinant subunit preparations and *in vitro* assemblies.
