# Supplementary material for: Bridge helix and trigger loop perturbations generate superactive RNA polymerases
Source: J Biol. 2008 Dec 2;7(10):40. doi: 10.1186/jbiol98 (PMC2776397; doi:10.1186/jbiol98)
Supplement: Additional file 21 — Bioinformatic analysis of intrinsic folding properties of bridge helices and trigger loops. [file jbiol98-S21.pdf]

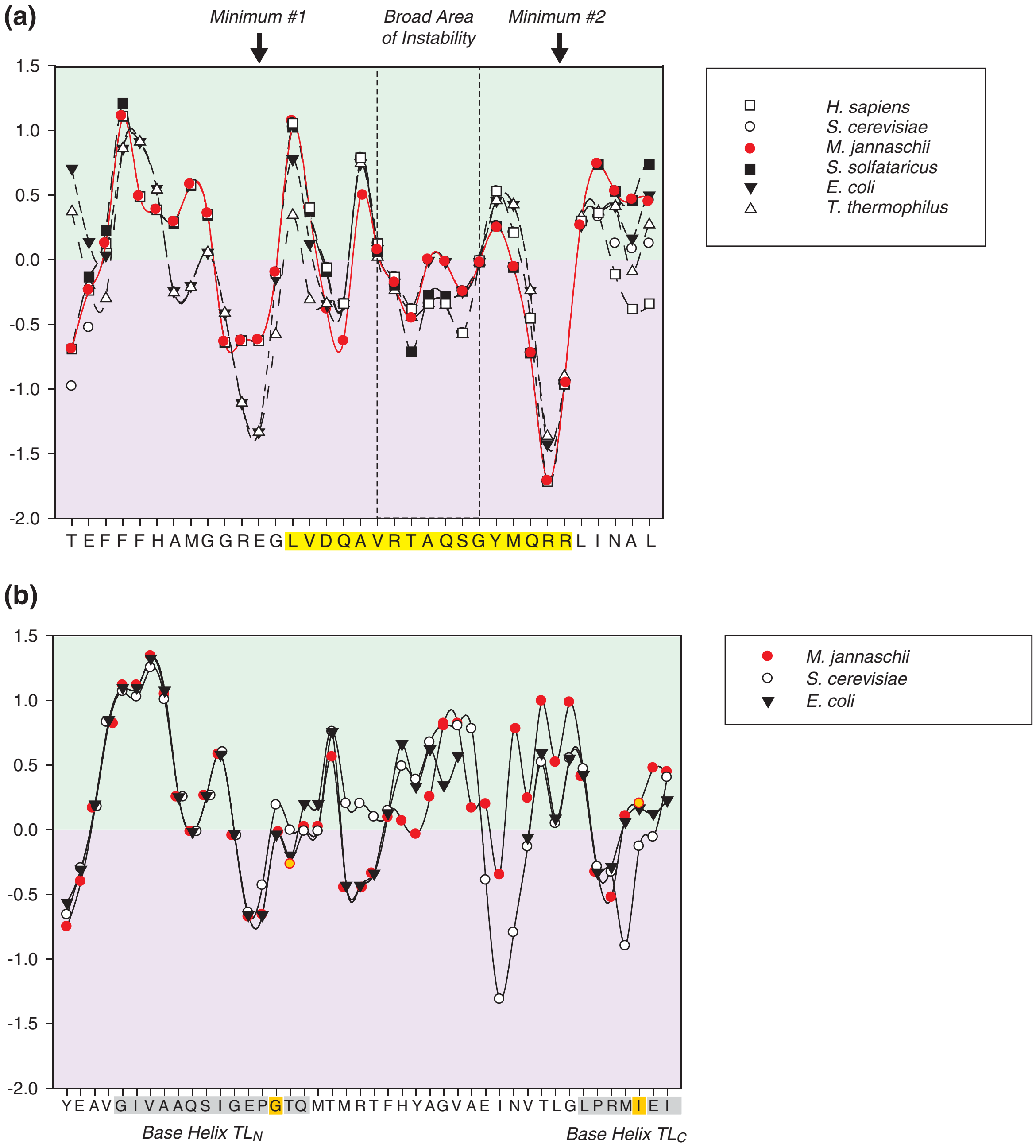

Additional Data File 21. Bioinformatic analysis of intrinsic folding properties of bridge helices and trigger loops

The 'FoldIndex' [36] was calculated for complete bridge helix and trigger loop domains and plotted. The sequence of the *M. jannaschii* domains are shown along the x-axis and the corresponding values are plotted with red symbols. Amino acid positions that are likely to be ordered have a FoldIndex > 0 and are shown in the light green regions. Residues that are probably part of disordered or structurally mobile domains have a negative FoldIndex and are shown in the purple region of the graphs. The symbols on the right hand side indicate the species of the analyzed sequences.

(a) FoldIndex plot of bridge helix domains. Many areas of the bridge helix have positive values, indicating stable folding. There are two sharp minima that are caused by two charged residues next to each other (R811-E812 and R829-R830). The largest consecutive sequence of residues with negative or near-zero FoldIndex values corresponds to an area (V819-G825) that has been identified as being capable of adopting alternative conformations [7,10,11].
There is also experimental evidence that R829 (located in 'Minimum #2') has an important role in stabilizing kinked versions of the bridge helix [10,11]. Note the high degree of similarity of the predicted FoldIndex values among the sequences including bacteria, archaea and eukaryotes.
(b) FoldIndex plots of trigger loop domains. Certain areas, especially base helix TLN, show a high degree of intrinsic stability. The borders between the base helices are either slightly unstable, or only weakly stable (G72 and I98, respectively; highlighted in orange). The bridge helix kink opposite these residues (see Figure 4c) is therefore likely to have a substantial influence on the folding properties of the central tip of the trigger loop domain. As expected, the trigger loop tip sequence (defined here as M75-G93) contains a mixture of intrinsically stable and unstable motifs capable of taking up a variety of structures. The stability profile of the flanking base helices is very similar in trigger loop domains from bacteria, archaea and eukaryotes, suggesting that the structural properties of these regions are highly conserved.
